# Rapeseed—An Important Oleaginous Plant in the Oil Industry and the Resulting Meal a Valuable Source of Bioactive Compounds

**DOI:** 10.3390/plants13213085

**Published:** 2024-11-01

**Authors:** Ancuţa Petraru, Sonia Amariei

**Affiliations:** Faculty of Food Engineering, Stefan cel Mare University of Suceava, 720229 Suceava, Romania; sonia@usm.ro

**Keywords:** circular economy, rapeseed meal, bioactive compounds, nutritive characteristics

## Abstract

Rapeseeds (*Brassica napus*), cultivated widely as a source of oil, generate substantial by-products after oil extraction. Unfortunately, rapeseed meal is considered a waste product and as such is discharged into environment as compost or used as animal feed. However, this meal is rich in bioactive compounds (proteins, minerals, fibers and polyphenols), indicating its potential for the development of value-added products. The meal shows a higher content of minerals, total dietary fibers and proteins. Rapeseed meal contains a proportion of oil rich in polyunsaturated fatty acids, predominately linoleic and α-linolenic acid. The amino acid proportion in the meal is higher than that in the seeds and contains essential amino acids, predominately valine. The analyses show the presence of valuable components in the cake, which makes it suitable for use in obtaining value-added products.

## 1. Introduction

Nowadays, consumers are requesting functional foods rich in nutrients and bioactive compounds with beneficial health effects [1]. Moreover, the growing human population leads to the necessity for sustainable food production [2]. Thus, by-products and residue resulting from the food industry that still contain high nutritive value are valorized and incorporated into the industrial chain, ensuring the circular economy principles [3].

Oilseeds are crop plants whose edible oil is suitable for human consumption. After soybean, rapeseed is the second most cultivated crop, surpassing peanut, sunflower and cottonseed [4]. These seeds possess advantages such as easy cultivation, good environmental sustainability and rich nutritional qualities [5]. Rapeseed is cultivated the most in Germany and France, followed by Russia, Pakistan, Canada, Australia, China and India [6,7].

Rapeseed meal is the biomass that remains after oil extraction. The oil can be extracted by two traditional methods: by using a solvent or a mechanical press (hot or cold pressing) [8]. These by-products are used primarily as animal feed, but they contain bioactive compounds with numerous health benefits for humans (anti-tumoral, -viral, -bacterial and -mutagenic abilities) [9]. Their nutritional composition depends on the extraction method, variety and the growing conditions of seeds [10].

The application of rapeseed meal is limited by its content in anti-nutritional factors, such as glucosinolates, sinapine and its derivates. These factors can be treated with physical, chemical (through the use of ethanol, methanol or acetone), biological (fermentation) and crop breeding methods [11]. The simplest method for decreasing the content of glucosinolates by up to 94% in rapeseed meal is its immersion in water (1:6) for 15–20 min. This treatment also improves the flavor and palatability of the meal [8].

Possibilities for valorizing the meal have been presented in the literature. The conventional methods include incineration, using it as animal feed, composting and biofuel conversion [12]. Increased awareness of environmental issues has led to new valorization methods, namely recovery of valuable components to create new value-added products [13,14] and the production of functional ingredients [10], new products and biopolymer films [15].

Meal is potentially a source of phenolic compounds [16]. Target compounds can be isolated by ultrasound-assisted extraction (UAE), a method that uses acoustic energy and an extraction solvent [17]. Ultrasound waves induce disruption of the cell walls (according to a dynamic process called cavitation) that facilitates the transfer of some compounds into the extraction medium. Moreover, a localized heating effect enhances the extraction process [5].

**Figure 1:**
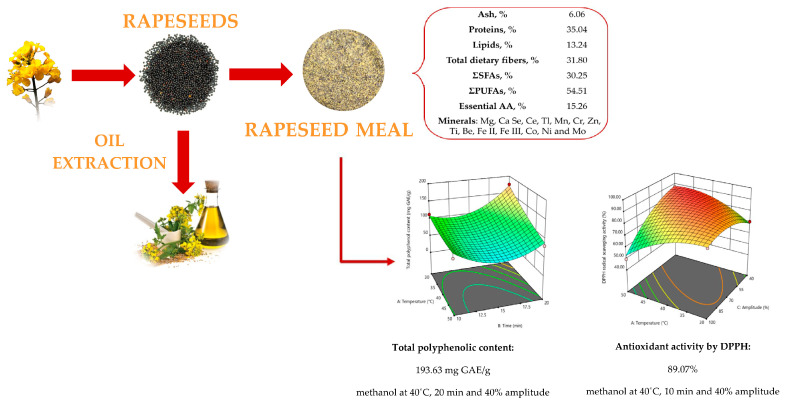
Rapeseeds and meal.

The objective of this study was focused on a nutritional, functional and safety assessment of rapeseeds, as well as the resulting meal after oil cold pressing extraction. The efficiency of different ultrasound-assisted treatments and solvents in terms of the total phenolic content and antioxidant activity of the rapeseed meal was also investigated (Figure 1).

## 2. Materials and Methods

### 2.1. Sample Description

Rapeseeds (RSs), rapeseed meal (RSOC) and rapeseed oil (RO) were purchased from an oil factory OLEOMET, in Romania. The seeds were manually cleaned to separate foreign materials, while the cake was ground and sieved (Retsch Vibratory Sieve Shaker AS 200 basic, Haan, Germany) to below 400 µm.

### 2.2. Raw Material Safety

The safety of the cake was demonstrated by the following analyses: water activity, spectroscopic methods and the ELISA method.

The water activity index (aw) was measured using an AquaLab 4TE water activity meter (Meter Group, Pullman, WA, USA).

The ELISA (enzyme-linked immunosorbent assay) method was performed using kits provided by ProGnosis Biotech S.A. (Larissa, Greece). The samples were analyzed for their content of zearalenone, ochratoxin A, aflatoxin B1 and deoxynivalenol [18].

Mineral elements were determined using ICP-MS (Agilent Technologies 7500 Series, Santa Clara, CA, USA) to highlight possible heavy metal contamination of the samples.

### 2.3. Nutritive Composition

The seeds and meal were investigated for their moisture, protein, ash, lipid and total dietary fiber content.

Their moisture was determined with a gravimetric method (ISO 665:2020 [19], AOAC935.29 [20]) by drying (at 105 °C) the samples in a laboratory oven (Zhicheng Analysis Instruments, Shanghai, China) until they reached a constant mass.

Protein content was determined by the Kjeldahl method (AOAC 950.48 [21]) with a conversion factor of 5.88.

The ash was determined by calcining 5 g of a sample at 550 °C for 6 h according to AOAC method 923.03 [22].

The fat content was determined using an automated Soxhlet extraction system with petroleum ether as the solvent (ISO 659:2020 [23], AOAC 920.39 [24]).

The total dietary fiber was determined according to AOAC 985.29 [25] using the Megazyme assay kit (Megazyme, Wicklow, Ireland).

The carbohydrate and energy values were calculating by applying Equations (1) and (2):

Carbohydrates (%) = 100 − (moisture + ash + protein + fat + fiber)(1)

Energy value (Kcal/100 g) = (4 × protein) + (9 × fat) + (4 × carbohydrates) + (2 × fibers)(2)

### 2.4. Physical Properties of the Rapeseeds

#### 2.4.1. Shape and Spatial Dimensions

Five randomized groups of whole seeds (100 pieces) were weighed to an accuracy of 0.1 mg using an analytical balance (PARTEN AS 220.R2, Radwag, Bucharest, Romania).

The seeds’ dimensions (length—L; width—W; thickness—T) were measured using a digital caliper (VOREL 15240, Wrocław, Poland, 0.003 mm accuracy).

The geometric mean diameter (D_g_), sphericity (ψ), surface area (S), projected area (A_p_) and volume (V) of the seeds were determined through comparison to a sphere using the following relationships [2]:

(3) $$D_{g}(mm)=\sqrt[{3}]{L\times W\times T}$$

ψ = Dg/L(4)

S (mm^2^) = *Π* × Dg^2^ (5)

(6) $$A_{p}\;({\mathrm{mm}}^{2})=¾\;\times \;L\times W$$

V (mm^3^) = W × L × T × φ (7)

#### 2.4.2. Gravimetric Properties

Bulk density (p_b_) was measured by filling a container with a 250 mL (V) capacity from a falling height of 150 mm and weighing the content (M), while the true density (p_t_) was determined using a pycnometer by recording the volume of toluene (M_T_; p_toluene_ = 0.867 g/mL) displaced after the immersion of a known quantity of seeds (M_s_) [26].

p_b_ (kg/m^3^) = M/V (8)

p_t_ (kg/m^3^) = (M_s_ × p_toluene_)/M_T_ (9)

Porosity (Φ) was calculated according to Equation (10):

Φ (%) = ((p_t_ − p_b_)/p_t_) × 100 (10)

### 2.5. Color and Functional Properties of the Meal

The color of the press cakes was measured using a CR-400 colorimeter (Konica Minolta, Tokyo, Japan) and the CIELAB system. The L* coordinate measures brightness (0 for black and 100 for white). A negative value for the a* coordinate indicates intensity of the color green, and a positive value indicates intensity of the color red. Parameter b* varies between −100 (indicating intensity of the color blue) and +100 (indicates intensity of the color yellow).

The water/oil holding capacity (WHC/OHC) was measured according to Omowaye-Taiwo et al. [27] with minor modifications: 1 g of the sample and 10 mL of distilled water/corn oil was placed into centrifuge tubes. These were kept at room temperature for 30 min and then centrifuged at 7000 rpm for 20 min. The results were expressed as grams of water/oil absorbed per gram of the sample.

The bulk density was analyzed using a volumetric method. Thus, 5 g of the sample was placed into a 100 mL cylinder, and it was gently tapped 20 times. Values were calculated as the ratio of the sample weight to the sample volume [28].

Least gelatinization concentration (LGC) was determined according to Marasingheand Rani [29,30] with some modifications. Suspensions of the samples and distilled water from 2% to 20% (*w*/*v*) were prepared in centrifuge tubes. The tubes were heated for 1 h in a boiling water bath and then rapidly cooled in water at 4 °C for 3 h. When inverting the tube, the concentration of the sample that does not fall or slide is considered the LGC.

Powder wettability was estimated using the method described by North et al. [31]. In brief, 2 grams of cake was transferred into a beaker containing 80 mL of distilled water. The behavior of the powder on the water’s surface was observed immediately after its addition. After 30 min of observation, the suspension was stirred fast enough to form a vortex that reached the bottom of the beaker. The contents were agitated for one minute. The degree of wetting was recorded as excellent, good, satisfactory or poor depending on time and the dispersion behavior.

Emulsion capacity (EC) and stability (ES) were analyzed according to the methods proposed by Rani [30] and Iyenagbe et al. [32]: First, 30 mL of a 0.5%suspension was mixed with 10 mL of corn oil. The emulsion was homogenized and immediately transferred into a graduated cylinder (50 mL) to read the height obtained. Emulsion stability was determined by heating the cylinder for 30 min at 80 °C. The final height of the emulsion was read.

(11) $$EC\;\left(\%\right)=\frac{Oil\;layer\;height}{Total\;suspension\;height}\times 100$$

(12) $$ES\;\left(\%\right)=\frac{Final\;suspension\;height\;after\;heating}{Initial\;suspension\;height}\times 100$$

Foam capacity (FC) and stability (FS) were determined using the method proposed by Naczk et al. [33], with some modifications. A suspension of 3 g of meal and 100 mL of distilled water was homogenized for 5 min at 1600 rpm. The mixture was immediately transferred into a 250 mL graduated cylinder, and the foam volume was noted. FS was determined by the decrease in the foam volume as a function of time (20, 40, 60 and 120 min). The results were calculated using the following equations:

(13) $$FC\;\left(\%\right)=\frac{volume\;after\;agitation-volume\;before\;agitation}{volume\;before\;agitation}\times 100$$

(14) $$FS\;\left(\%\right)=\frac{\;foam\;volume\;after\;a\;set\;time\;}{initial\;foam\;volume}\times 100$$

### 2.6. Seed and Meal Comparisons

#### 2.6.1. Fatty Acids

The seeds and oleaginous cake were analyzed to highlight the composition in fatty acids (FAMEs). Their derivatization was performed according to the following procedure: 30 mg of the oil sample was mixed with 2 mL of isooctane. The solution was then subjected to transesterification through the addition, under vigorous stirring, of 200 µL of potassium hydroxide (2 mol/L methanolic solution). The resulting organic phase was mixed with sodium sulfide, and the supernatant was collected [34].

FAME separation was performed using a gas chromatograph (GC-FID, Agilent Technologies, 6890N GC, Wilmington, DE, USA) and using a stationary polyethylene glycol DB-WAX capillary column (30 m length, 0.25 mm inner diameter and 0.25 µm thickness). The initial temperature of the oven was set to 60 °C for 1 min, it was increased from 60 °C to 200 °C (by 10 °C/min) and held for 2 min and then increased again (by 5 °C/min) from 200 °C to 220 °C and held for 20 min. The flow rates were maintained at 40, 450 and 30 mL/min for hydrogen, air and helium respectively. The injection port and the detector temperatures were 250 °C. FAME identification and quantification were performed by comparing their retention times with those of the standard mixture. Fatty acid composition was expressed as µg/mL and as the relative level (%) of fatty acid composition. Each determination was performed three times [35].

#### 2.6.2. Free Amino Acids

A mixture of the sample (0.7 g) and 15% trichloroacetic acid (6 mL, TCA) was made. The mixture’s pH was adjusted to 2.2 ± 0.05 with sodium hydroxide solution (1–4 M), and then the volume was brought to 10 mL exactly with acid. The solution was centrifuged for 5 min at 3000 rpm, and the supernatant was filtered (trough 0.45 µm). The solution containing amino acids was analyzed using the EZ:faast GC-MS kit (Phenomenex, Torrance, CA, USA) [36].

The analysis was performed using a gas chromatograph coupled with a mass spectrometer (Shimadzu, Kyoto, Japan) and a ZB-AAA column(10 m × 0.25 mm). The analysis time was 10 min, and the injected volume was set to 0.002 mL. The initial temperature of the oven was 110 °C, which was then increased to 320 °C and held for three minutes. The conditions for the mass spectrometer were 200 °C for the ion source and 320 °C for the interface. The amino acid mixture solutions included in the kit mentioned above were used for calibration [37].

#### 2.6.3. Mineral Content

The ash resulting after calcination was dissolved with 65% nitric acid (0.73 mL), and the solution was brought to a 50 mL volume with deionized water [2]. The minerals (Li, Be, Mg, Ti, Tl, Co, As, Ca, Cd, Cr, Ce, Cu, Hg, Fe, Mn, Ni, Pb, Se, Sr, Sb, Mo, V and Zn) were estimated with an inductively coupled plasma mass spectrometer (ICP-MS, Agilent Technologies, Santa Clara, CA, USA).

### 2.7. Fourier Transform Infrared-Attenuated Total Reflectance (FTIR-ATR)

The meal powders were analyzed using FTIR-ATR. Their spectra were recorded using a Nicolet iS20 spectrometer (Thermo Scientific, Karlsruhe, Germany) equipped with an attenuated total reflectance accessory and a diamond crystal. The spectra were collected within the range of 400 cm^−1^ to 4000 cm^−1^ at a 4 cm^−1^ resolution and using 32 scans. The spectra obtained were processed using OMNIC software (version 9, Thermo Scientific) [38].

### 2.8. Preparation of the Rapeseed Meal Extract

The application of different treatments (in terms of choice of solvent, use of heat, agitation and time) caused a change in the content of total polyphenols (the TPC) and, implicitly, the free radical scavenging activity (DPPH). Evaluation of these changes was carried out using the software Design Expert 11 (Stat-Ease Inc., Minneapolis, MN, USA, trial version) through the response surface methodology using the Box–Behnken model. After studying the literature in the model, 4 factors were varied, and 3 replications were provided at the central point. Each factor was varied at 3 levels (Table 1) as follows: temperature (A: 30, 40 and 50 °C), time (B: 10, 15 and 20 min), amplitude (C: 40, 70 and 100%) and solvent (D: 80% methanol, water and 80% ethanol) [39,40,41].

The extracts were made by sonicating a mixture of defatted meal and the solvent (at a ratio of 1:20) in an ultrasonic bath (Elma Transsonic TI-H15, Singen, Germany), varying the parameters of temperature, time and amplitude and keeping the frequency constant at 45 Hz.

Ultrasound energy is the product of frequency and amplitude. If the frequency is constant, the only possibility for changing the ultrasonic energy is changing the amplitude of the sinusoidal signal [42].

### 2.9. Total Phenolic Content (TPC)

To determine the TPC, 0.2 mL of the extract prepared as described previously was mixed with 2 mL of Folin–Ciocâlteu reagent (diluted 1:10) and 1.8 mL of 7.5% sodium carbonate. The mixture was left at room temperature for 30 min, and the absorbance was read at a 750 nm wavelength using an UV-VIS NIR spectrophotometer (Shimadzu Corporation, Kyoto, Japan) [43]. Gallic acid was used to generate calibration curves at concentrations of 10–500 mg/L. The regression coefficient was 0.99658, while the equation y = 0.00484x + 0.16058. The sample was analyzed in duplicate.

### 2.10. DPPH Radical Scavenging Activity

For determination of the radical scavenging activity, 0.3 mL of the extract prepared as described previously was mixed with 2.7 mL of 0.1 mM DPPH reagent (prepared in methanol). The mixture was shaken and kept at room temperature for 30 min [44]. The absorbance was read at a 517 nm wavelength using an UV-VIS-NIR spectrophotometer (Shimadzu Corporation, Kyoto, Japan). The results were expressed as the percentage of DPPH discoloration according to Equation (8):

(15) $$Scavenging\;effect\left(\%\right)=\frac{{Absorbance}_{DPPH}-{Absorbance}_{sample}}{{Absorbance}_{DPPH}}\times 100$$

### 2.11. Statistical Analysis

All the results are presented as mean ± standard deviation. The nutritional and functional analyses for the rapeseeds and meal were performed in triplicate. Meanwhile, the amino acid, fatty acid and mineral analyses were performed in duplicate. A sample of 500 seeds was used for physical characterization of the seeds. The values obtained were processed using Excel-Stat software (trial version). Differences between the samples were established using an analysis of variance (ANOVA) and using Tukey’s test at a 95% confidence level. To determine the chemical differences between the seeds and meal, Student’s *t*-test was performed.

## 3. Results and Discussion

### 3.1. Meal Safety

The value obtained for water activity was low (0.4153), lower than 0.6, which does not allow for the development of molds, yeasts or bacteria.

The mineral analysis using ICP-MS showed the absence of heavy metals such as lead, mercury and cadmium.

The results for the studied mycotoxins fell within the legal stability limit allowed by the European Union, and these are presented in Table 2.

### 3.2. Physical Properties

The rapeseeds showed variations in their physical properties (Table 3), between 1.70 and 2.88 mm for length; between 1.60 and 2.73 mm for width; between 1.22 and 2.49 mm for thickness; between 1.61 and 2.55 for the geometric mean diameter; between 3.78 and 15.01 mm^3^ for volume; and between 8.13 mm^2^ and 20.36 mm^2^ for the surface area. The average sphericity was 91%, which indicated that the seeds are spherical and easily roll on structural surfaces. The values for bulk density, true density and porosity were 694.20 kg/m^3^, 1070.17 kg/m^3^ and 37.50%, respectively. 

Compared to the Turan variety, our seeds are longer but have the same thickness and width [38]. The opposite was observed for the varieties Elvis and Capitol, which are longer and narrower, and for the varieties Jetneuf and Samurai, which are longer and larger than those investigated in this study [45,46].

The results found were consistent with the best claims by other authors (L = 1.52–2.96 mm; W = 1.47–2.68 mm; T = 0.99–2.01 mm; D_g_ = 1.63–2.23 mm; Ψ = 0.82–0.97; mass of 1000 grains = 2.85–6.36 g; S = 1.67–15.7 mm^2^; Φ = 1.62–10.07 mm^3^; bulk density = 585.1–738.8 kg/m^3^; true density = 1091.3 kg/m^3^; M = 0.0038–0.0065 g; A_p_ = 3.60–4.67 mm^2^) [45,46,47,48,49,50,51,52].

The correlation coefficients (Table 4) show the relationships between the dimensional properties of the seeds. All correlations found, except D_g_/Ψ and Ψ/S, were significant at *p* < 0.5%. The parameters L, W and T showed moderate positive correlations, indicating a certain dependence between them (long seeds are also the widest, r = 0.676, and wide seeds are also the thickest, r = 0.543).

The influence of mass was investigated by calculating the correlation coefficients for all combinations. Although significant values were found at *p* < 0.05, they all indicated weak linear correlations (r = 0.088–0.167), indicating independence between them. Therefore, longer, wider and denser seeds are not necessarily the heaviest.

The physical properties (length, width and thickness) were integrated into the formula for calculating the geometric mean diameter, surface, sphericity and volume. For this reason, strong positive correlations between these formulas were easy to achieve.

### 3.3. Nutritional Composition of the Seeds and Meal

#### 3.3.1. Chemical Composition

The nutritional composition (Table 5) of the whole seeds and meal after cold pressing extraction presented significant differences (*p* < 0.05). Except for fat content, all the parameters (moisture, ash, proteins, fibers and carbohydrates) were increased in the meal. This may be related to the cold extraction method, which leaves a significant content of oil that increases the nutritional value of the meal. The nutritional values, except for the moisture, were significantly different at *p* < 0.05.

Various studies on the nutritional properties of rapeseed meal (after cold extraction) have shown that the moisture, ash, proteins, lipids, total dietary fiber and carbohydrates ranged between 6 and 10.8%, 4.19 and 19.7%, 14.03 and 40.1%, 5.14 and 23.1%, 5.5 and 20.11% and 8.35 and 48%, respectively [8].

Regarding the seeds, the nutritional parameters found in the literature vary as follows: moisture: 1.96–6.4%; ash: 3.8–4.84%; proteins: 20.85–25.7%; lipids: 38.80–40.60% [45].

Due to the high fat content (35.22%), which has the highest energetic contribution (9 kcal/g), the seeds have a higher caloric value. Similar values have been found in the literature (559.60–572 kcal/100 g in rapeseeds and 348,2 kcal/100 g in meal [53,54])

#### 3.3.2. Mineral Composition

Oilseeds are a valuable source of mineral elements (Table 6) that have an important role for human health (with involvement in basic biological mechanisms). The major macroelements identified in the rapeseed meal were Mg and Ca, which represented 73.60% of the total ash content (6.06%). Among the microelements, Se showed the highest concentration followed by Ce, Tl, Mn, Cr, Zn, Ti, Be, Fe II, Fe III, Co, Ni and Mo. After oil extraction, except Tl and Li, all the minerals increased. The element Li was the only element not present in the meal.

For the meal, the following values were found in the literature: Cu: 3.42–10.00 mg/kg; Fe: 69.23–159 mg/kg; Mn: 37.77–62.26 mg/kg; Mo: 0–1.5 mg/kg; Zn: 45.00–71.00 mg/kg; Se: 0–1.22 mg/kg; Ca: 5750–7330 mg/kg; Mg: 3500–4690 mg/kg [55,56].

A total of 17 elements were found in the seeds (Mg > Ca > Se > Tl > Ce > Mn > Cr > Zn > Cu > Ti > Be > Fe II > Co > Fe III > Li > Ni > Mo) and oil (Tl > Ce > Mg > C > Se > Li > Mn > Cu > Be > Zn > Mo > Ni > Ti > Fe II > Co > Fe III). In the seeds, the macroelements represented 71.83% of the total ash content (4.26%).

The values found for the seeds were similar to those found by other authors: Ca: 3560–29,955 mg/kg; Mg: 2483.00–3543 mg/kg; Fe: 30.93–195 mg/kg; Mn: 15.56–96.30 mg/kg; Cu: 1.25–21.25 mg/kg; Zn: 21.46–88.90 mg/kg; Cr: 0.80–1.65 mg/kg; Ni: 1.85–2.40 mg/kg [53,57].

A comparative study of the mineral composition showed that the majority of the elements were increased in the meal, and only the content of Tl and Mg was decreased. Li was the only element not found in the meal because it passed into the extracted oil.

In the oil Mg, Ca, Ce and Tl were present to high values, while the other elements were present in proportions ≤ 2%. The values found in the literature for rapeseed oil were lower than those found in our study [57].

#### 3.3.3. Fatty Acids

The remaining oil provides its nutritional and health properties to the partially defatted powder, especially in increasing the content of unsaturated fatty acids (Table 7). A total of 31 fatty acids were determined, of which 15 were saturated (SFAs), 7 were monounsaturated (MUFAs) and 9 were polyunsaturated (PUFAs). The difference between the samples was significant at the 95% confidence level.

The seed oil contained 24.40% saturated fatty acids (SFAs), 22.75% monounsaturated fatty acids (MUFAs) and 52.87% polyunsaturated fatty acids (PUFAs). The most predominant fatty acids identified in the seeds were linoleic and linolelaidic acids, followed by palmitic, oleic and elaidic acids. Eicosadienoic and myristoleic acids were found in small amounts. A lower amount of stearic acid was found than palmitic acid.

The rapeseed meal contained 30.25% SFAs, 15.24% MUFAs and 54.51% PUFAs. Compared to the seeds, in the SFA classes of the meal, an absence of palmitic, tricosanoic and lignoceric acids was observed, as was the presence of heptadecanoic acid. Linoleic, linolelaidic, heptadecanoic and α-linolenic acids were the most predominant fatty acids present in the rapeseed meal. Rapeseeds and meal were found to be poorer in SFAs and PUFAs in the literature [53,56,57].

Excessive consumption of saturated fatty acids has a negative effect on human health, especially on the cardiovascular system. In this regard, both investigated samples were rich in UFAs. Moreover, PUFAs were more abundant than MUFAs in both samples, and these findings indicate their importance for health. PUFAs improve fluidity and permeability through cellular membranes and reduce the risk of cardiovascular diseases and autoimmune disorders [35].

PUFAs n-6 and n-3 are essential fatty acids that cannot be produced by the human body and must be taken in our diet [2]. In a healthy balanced diet, the optimal n-6/n-3 ratio ranges between 1:1 and 5:1. However, the modern diet includes high consumption of PUFAs n-6 (at ratios of 10:1 and 20:1), which increases the risk of developing inflammatory diseases, such as obesity [58]. The group of PUFAs n-3 investigated in this study was represented by α-Linolenic acid, cis-11,14,17-eicosatrienoic acid and cis-4,7,10,13,16,19-docosa-hexanoic acid. On the other hand, the group of PUFAs n-6 was represented by γ-Linolenic acid, Linoleic acid + Linolelaidic acid, cis-11,14-eicosadienoic acid + cis-8,11,14-eicosatrienoic acid, arachidonic acid and cis-4,7,10,13,16,19-docosahexanoic acid (C22:2, n-6).

The n-6/n-3 PUFA ratio in the meal was 3.17. This value was lower than that in the seeds (34.29), indicating a positive nutritional profile and a beneficial effect on cardiovascular risk factors [59]. The values found were much lower than those found in the literature, which ranged between 5.46 and 7.08 [55].

#### 3.3.4. Amino Acids

The content of protein in the rapeseed meal was increased the most (35.04% for the meal vs. 24.29% in the seeds). Considering this increase, an evaluation of protein quality by determining the amino acid (AA) profile is necessary (Table 8).

The meal had a significantly (*p* < 95%) higher content of total AAs (38,795.21 nmol/g in seeds vs. 45,710.55 nmol/g in meal). The most abundant amino acid found in the seeds was glutamic acid, followed by aspartic acid, asparagine, proline, alanine and glycine. In meal, the highest AA content found was that for serine, followed by glutamine, glycine, alanine and asparagine. The lowest contents of AAs identified in the seeds and the meal were those of α-aminobutiric acid, β-aminobutiric acid, leucine and proline/hydroxyproline.

For all the AAs, relative percentages were calculated, with which the total percentage of essential/non-essential AAs was calculated. The essential AAs investigated were valine, leucine, isoleucine, threonine, methionine, phenylalanine, histidine and tryptophan. Leucine, isoleucine and valine are named branched-chain AAs. They must be obtained through our diet and cannot be synthetized by the body. These AAs are the building blocks for the synthesis of proteins that play an important role in the body’s energy metabolism [1].

In the meal, the essential AAs represented 15.26%, while in the seeds, they represented 16.01%. Valine was the major AA present in both the seeds and the meal.

In seeds, the pattern of the other essential AAs was methionine > histidine > threonine > lysine > tryptophan > isoleucine > phenylalanine > leucine.

Threonine was the only essential AA that was not present in the meal. In decreasing order, the other essential AAs present in the meal were histidine > methionine > lysine > isoleucine > tryptophan > leucine. The values found are in the range found in the literature (13.13–19.26) [60,61,62,63].

### 3.4. Functional Properties

Functional properties are important in food product manufacturing, transportation, storage and stability [64]. The functional properties investigated were bulk density (BD), water holding capacity (WHC), oil holding capacity (OHC), emulsion capacity/stability (EC/ES), foam capacity/stability (FS/FC) and least gelatinization concentration (LGC). The values found for rapeseed meal were 0.5942 g/mL, 2.92 g/g, 1.29 g/g, 25.65%, 93.33%, 6.47% and 16%, respectively, for BD, WHC, OHC, EC, ES, FC and LGC (Table 9).

The OHC represents the amount of fat absorbed by the non-polar side of the protein chain, while the WHC represents the amount of water absorbed by the polar side of the protein chain. The interaction of water and oil with rapeseed meal is important in food formulations due to its influence on its taste and texture [65]. The fat acts as an enhancer of mouthfeel and a flavor retainer [66]. The values found in the literature for OHC and WHC were 0.90–7 g/g and 1.1–6.2 g/g, respectively [4,33,67,68,69,70].

BD is the property that describe a powder’s weight and depends on the size, shape and state of compaction of the powder particles [30,71]. The results obtained were in accordance with those found in the literature (0.498–0.726 g/mL) [72,73,74].

EC measures the amount of protein that mixes with oil. An emulsion is a two-phase system that includes immiscible liquids (water and oil) [66]. The emulsion stability measures the amount of water released by an emulsion over time [2]. These parameters depend on proteins’ flexibility and hydrophobicity, which improve the molecular anchoring of the oil/water interface and thus more stable emulsions are obtained [30]. The values obtained were in the range found in the literature (20.00–56.60% for EC and 70–108% for ES) [33,67,68,70].

LGC represent the minimum concentration of powder necessary to form a gel. The process of gelation implies the transformation of a viscous liquid into a three dimensional viscous–elastic matrix due to the swelling of proteins and starch through ordered polymerization of the molecules by heating [64]. The concentration obtained in our study was 16%. Similar values was found in the literature (14.90–15.70%) [68].

Regarding the color, the rapeseed meal powder was dark (a low lightness value) with a reddish and yellowish tone (values for a* and b* were positive).

**Figure 2:**
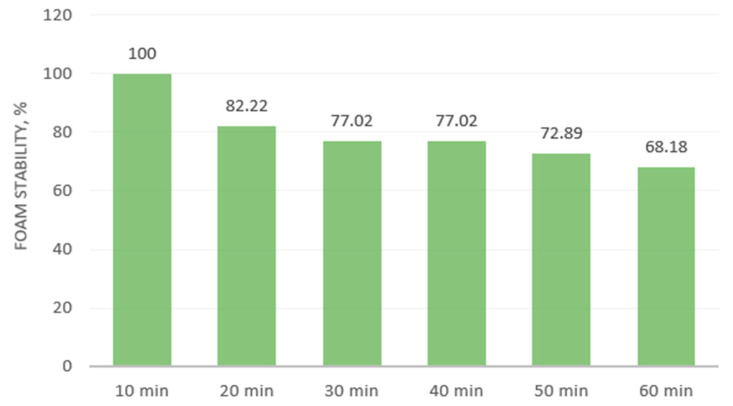
Foaming stability of rapeseed meal within 1 h.

The FC is a measure of the interfacial area formed by a protein during foaming [75]. The rapeseed meal showed a low foaming (6.47%) capacity, but the foam had high stability. The foam decreased from 100% to 68.18% within an hour (Figure 2).

The powder wettability (depending on time and the dispersion behavior) was recorded as good. On contact with water, the meal powder gradually moistens. Part of the powder is dispersed in the liquid, and the other is deposited at the bottom of the Berzelius beaker. After a few seconds, everything fades to the background. After half an hour, all of the powder particles settle at the bottom of the Berzelius beaker. By vortexing, the sample is dispersed through the liquid. 

### 3.5. Qualitative Analysis of Main Functional Groups

Fourier Transform Infrared Attenuated Total Reflectance (FTIR-ATR) can be considered a green, rapid and innovative method for characterizing samples. This method furnishes the chemical and biochemical characteristic substances in a sample by recording their molecular vibration (torsion, bending and stretching of the chemical bonds).

**Figure 3:**
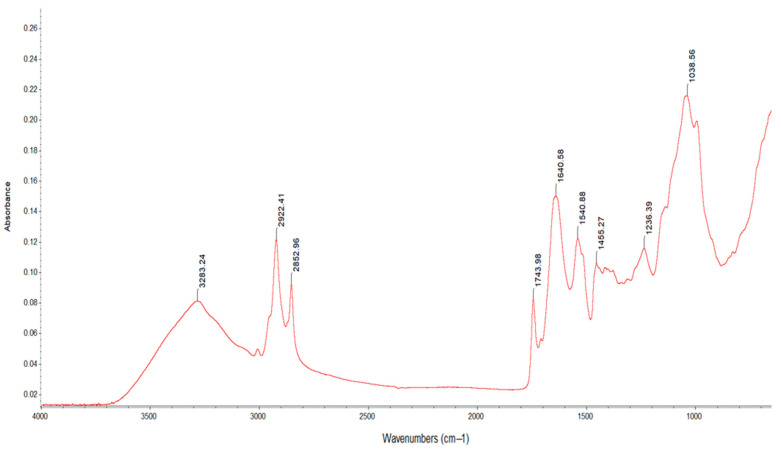
FTIR spectra for rapeseed meal.

The FT-IR spectra presented nine wavenumbers (Figure 3) in three different spectra zones, namely 4000–2500 cm^−1^ (the single-bond region), 2000–1500 cm^−1^ (the double-bond region) and 1500–600 cm^−1^ (the fingerprint region).

In the first region, there were three absorption wavenumbers, namely 3283.24 cm^−1^, 2922.41 cm^−1^ and 2852.96 cm^−1^, possibly attributed to the OH stretching modes of polysaccharides and/or cellulose [76] and asymmetric and symmetric stretching vibrations, mainly associated with the hydrocarbon chain found in fatty acids [77].

In the second region, the spectral band at 1743.98 cm^−1^ can possibly be attributed to the C=O bonds of the ester group. This is related to the presence of fatty acids and their carbohydrates, pectins and lignin [78]. The peaks at 1640.58 cm^−1^ and 1540.88 cm^−1^ were amidic bands I and II, respectively [79]; these result from the C=O stretching of amides (which involves the carbonyl stretching vibration of the peptide backbone) and bending variations in the N-H groups [56].

The fingerprint region was rich in peaks (from various stretching, bending, rocking, scissoring and torsional modes), providing information about the organic compounds. Unfortunately, due to its complexity, it is difficult to analyze [80]. The spectral band at 1455.27 cm^−1^ possibly corresponded to aromatic C-C stretching related to the presence of phenolic compounds, while the bending vibration of OH at 1236.39 cm^−1^ and the C-O stretching at 1038.56 cm^−1^ may have been related to polysaccharide substances [81,82].

### 3.6. Model Fitting and Statistical Analysis

The rapeseed meal extracts were obtained according to the information presented in Table 10. A total of 45 experiments were conducted with various combinations of independent variables (A, temperature, B, time, C, amplitude used for ultrasound-assisted treatment and D, solvent used for extraction). The experimental data recorded for the total polyphenol content and 2,2-diphenyl-1-picrylhydrazyl free radical scavenging activity are also presented in Table 10.

The ANOVA for the model fitting showed that the mathematic model chosen was statistically significant (*p* < 0.0001) and predicted the responses accurately. Total polyphenol content and antioxidant activity according to the DPPH data were fitted to a quadratic model, which explained 94% and 91% of the data variation, respectively.

For the experimental data, a multiple regression analysis was applied, obtaining a quadratic polynomial, in Equation (16), where R represents the responses (TPC and DPPH), x_0_ is the constant and b_1_-b_16_ are the coefficients of regression found in Table 11:

(16) $$R=b_{0}+b_{1}\times A+b_{2}\times B+b_{3}\times C+b_{4}\times D1+b_{5}\times D2+b_{6}\times AB+b_{7}\times AC\;+b_{8}\times AD1+b_{9}\times AD2+b_{10}\times BC+b_{11}\times BD1+b_{12}\times BD2+b_{13}\times CD1+b_{14}\times CD2\;+b_{15}\times A^{2}+b_{16}\times B^{2}+b_{17}\times C^{2}$$

The TPC ranged from 7.14 to 193.63 mg GAE/g (the maximum values were obtained using methanol at 40 °C, for 20 min and at a 40% amplitude), and the free radical scavenging activity ranged from 40.24% to 89.07% (the maximum values were determined using methanol at 40 °C, for 10 min and at a 40% amplitude).

The values found in the literature were 6.97–21.04 mg GAE/g for total polyphenol content and 6.59–69.08% for antioxidant activity [4,77,83,84]. The TPC and the antioxidant activity are dependent on the type of solvent used, its concentration, the granularity of the sawdust and the temperature. Teh et al. [83] investigated the effects of using different solvents with varying concentrations on the polyphenol content, obtaining high values using a mixture of solvents (methanol:acetone:water), followed by 80% acetone, 80% methanol and ethanol. Nandasiri et al. [84], obtaining high values for antioxidant activity, used 70% methanol and ethanol and high temperatures. The meal fraction particles (below 250 µm) led to the highest values for these two responses [4].

Phenolic acids and flavonoids are the main contributors to antioxidant activity. This correlation was demonstrated by Sepahpour et al. [85] for four different plants (turmeric, curry leaf, ginger and lemongrass), and generally, a high TPC was correlated with high DPPH values.

### 3.7. Total Polyphenolic Content

Temperature (A) had a significant negative effect on polyphenol content (*p* < 0.05), while time and amplitude (B and C) had a significant positive effect (*p* < 0.05 and *p* < 0.001, respectively). The highest positive effect was observed for the quadratic term of time (B^2^). From the equation, it can be observed that the interaction between temperature and time had the highest negative effect on TPC.

The variables temperature and amplitude had a greater positive effect on TPC when water was used as the extraction solvent. On the other hand, the variable time had a greater effect when the solvent used was ethanol. The combination of all variables had a negative effect on TPC for all the solvents used, and the strongest negative effect was observed for the combination of time and solvent (in this case ethanol), followed by that of temperature and time.

**Figure 4:**
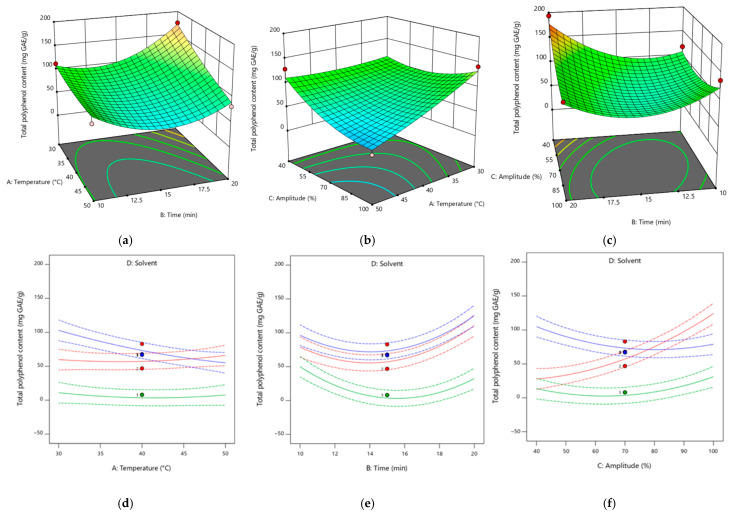
Response surface plots showing the effect of the extraction parameters and different solvents on the phenolic compounds. Pictures (**a**–**c**) are for the extraction in methanol. For pictures (**d**–**f**), the color green corresponds to ethanol, blue corresponds to methanol and red corresponds to water.

A 3D picture that shows the effects of different treatments on the TPC is presented in Figure 4.

### 3.8. DPPH Free Radical Scavenging Activity

Amplitude (C) had a significant (*p* < 0.05) negative effect on antioxidant activity, while temperature and time (A and B) had no significant effect. The largest positive effect was observed for the interaction between time and solvent2 (BD2-ethanol). On the other hand, solvent D2 (ethanol) had the greatest negative effect on antioxidant capacity.

Temperature had a greater positive effect on antioxidant activity when water was used as the extraction solvent. On the other hand, time and amplitude had a greater effect when the solvent used was ethanol. Only the combination of time and amplitude had a positive effect on antioxidant activity for all solvents used.

**Figure 5:**
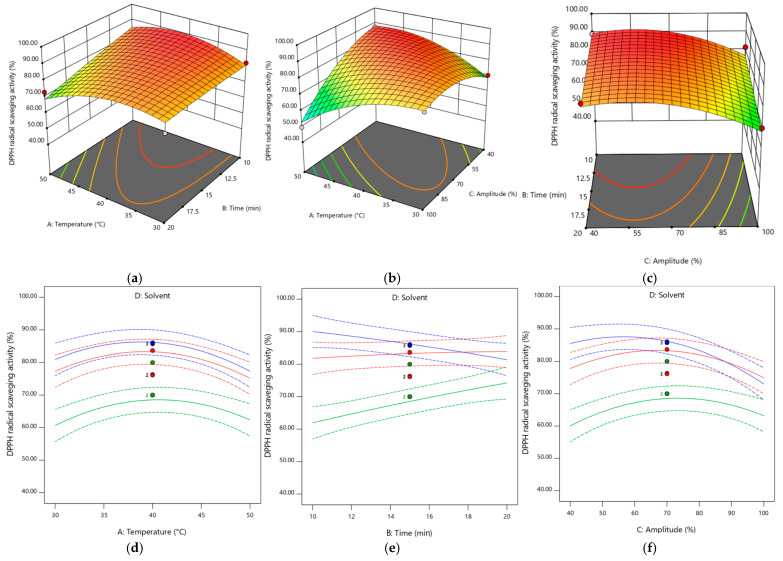
Response surface plots showing the effect of the extraction parameters and different solvents on the antioxidant activity. Pictures (**a**–**c**) are for the extraction in methanol. For pictures (**d**–**f**), the color green corresponds ethanol, blue corresponds to methanol and red corresponds to water.

The surface responses in Figure 5 show the effect of different factors on the evolution of antioxidant activity.

### 3.9. HPLC Analysis of Phenolic Compounds

The phenolic acids were determined for the extract with the highest TPC. The average concentrations found of the phenolic acids in the rapeseed meal are presented in Table 12.

The most abundant phenolic compound was vanillic acid, followed by myricetin, chlorogenic acid and caffeic acid. The concentration sum for the phenolic compounds detected accounted for 9272.28 mg per kg of rapeseed meal. This value is in the range found in the literature (410–21,602.9 mg/kg) [56,83,86]. The total obtained after summing the individual phenols was lower compared to the overall total obtained using the Folin–Ciocâlteu method because this reagent interacts not only with phenols but also with UFAs, AAs, carbohydrates, proteins and vitamins [43].

There are several studies in the literature regarding the phenolic components of rapeseed meal. Teh et al. [83] found gallic acid, p-coumaric, catechin, caffeic acid, epicatechin, ferulic acid, quercetin and luteolin. Meanwhile, Di Lena et al. [56] in addition found protocatechuic acid, 4-hydroxybenzoic acid, vanillic acid, chlorogenic acid, neochlorogenic acid, syringic acid, cryptochlorogenic acid, sinapic acid and cinnamic acid.

## 4. Conclusions

The increased popularity of the sustainability concept has led industrial production to develop new strategies for the efficient use of all resources without creating waste. Rapeseeds are grains used primarily for oil production. The extraction process generates large amounts of rapeseed meal, which is currently under-used.

The results of this study highlight the nutritional features of rapeseeds and meal after oil extraction. Cold-pressed rapeseed meal showed great potential for the formulation of various foodstuffs due to its high protein, mineral and dietary fiber contents.

The meal is considered safe to use because its mycotoxin content was in the allowed range and heavy metals were absent. Furthermore, its water activity index was low < 0.4, which means it does not allow for microorganism development.

Mainly, rapeseed meal is a valuable source of protein (35.04%), which includes essential amino acids (15.26%), especially valine. Rapeseed meal is rich in minerals such as Mg, Ca Se, Ce, Tl, Mn, Cr, Zn, Ti, Be, Fe II, Fe III, Co, Ni and Mo. Regarding its dietary fiber content, rapeseed meal can meet the consumer demand for fiber-rich food.

The meal also presented good water-holding and fat absorption capacities, emulsifying and foaming activities and stability. These important factors make rapeseed meal suitable for use in products in which hydration and viscosity improvements are necessary.

The total polyphenolic content and antioxidant activity values were affected by both the ultrasonic treatment and the solvent used. The best values were obtained when methanol was used for the extraction. Of all the parameters applied, the temperature applied had the most negative influence on TPC. The amplitude and time of ultrasonication positively influences both the TPC and the antioxidant effect of the rapeseed meal. Based on the mathematical models obtained, the extraction process can be optimized to obtain the maximum yield in terms of the total content of polyphenols with a high antioxidant activity.

Rapeseed meal is a low-cost, renewable resource rich in bioactive compounds. Due to its rich content in desirable nutrients, its use in the food industry is inevitable, not only for nutritional purposes but also due to its beneficial impact in terms of its textural and antioxidant qualities. It can be used as a co-product in the food industry for the manufacture of novel high-value-added products or supplements (through the extraction of proteins and dietary fibers). Other future directions for this valuable by-product lie in the realization of edible and biodegradable food packaging materials.

## Figures and Tables

**Table 1:** Experimental values (coded and actual).

Factors	Values		
	−1	0	+1
Temperature (°C) (A)	30	40	50
Time (min) (B)	10	15	20
Amplitude (%) (C)	40	70	100
Solvent (D1)	Methanol	Ethanol	Water
Solvent (D2)	Methanol	Water	Ethanol

**Table 2:** Incidence of mycotoxins in rapeseed meal.

Sample	Limit of Detection µg/kg	Limit of Quantification µg/kg	Results µg/kg	Maximum Limit 2006/576/EC µg/kg
*Zearalenone*	10	15	25.17 ± 2.83	2000
*Ochratoxin A*	0.5	1.5	3.88 ± 0.62	50
*Aflatoxin B_1_*	0.3	0.7	<LOQ	10
*Deoxynivalenol*	0.011	0.042	0.207 ± 0.035	0.9

**Table 3:** Shape, dimensional and gravimetric parameters for the seeds; n = 500.

Sample	Range	Average
**Shape and dimensional parameters**		
L, mm	1.70–2.88	2.24 ± 0.20
W, mm	1.60–2.73	2.06 ± 0.17
T, mm	1.22–2.49	1.85 ± 0.18
D_g_, mm	1.61–2.55	2.04 ± 0.15
Ψ, -	0.75–1.09	0.91 ± 0.05
V, mm^3^	3.78–15.01	7.87 ± 1.84
S, mm^2^	8.13–20.36	13.14 ± 1.96
A_p_, mm^2^	2.24–5.79	3.64 ± 0.57
**Gravimetric parameters**		
M, g	0.0028–0.0099	0.0057 ± 0.001
mass of 1000 grains, g	5.29–5.47	5.37 ± 0.08
p_b_, kg/m^3^	686.05–698.56	694.20 ± 3.36
p_t_, kg/m^3^	898.82–1176.68	1070.17 ± 98.57
φ, %	22.81–41.25	37.50 ± 2.33

L, W, T, M, D_g_, Ψ, V, S, A_p_—length, width, thickness, mass, geometric mean diameter, sphericity, volume, surface area and projected area of sunflower seeds; p_b_—bulk density; p_t_—true density; φ—porosity.

**Table 4:** Correlation between the physical properties of rapeseeds; n = 500.

Variables	M	L	W	T	D_g_	*Ψ*	V	S	A_p_
**M**	1								
**L**	0.167 *	1							
**W**	0.088 *	0.676 *	1						
**T**	0.009	0.365 *	0.543 *	1					
**D_g_**	0.100 *	0.803 *	0.877 *	0.800 *	1				
**Ψ**	−0.133 *	−0.529 *	0.116 *	0.525 *	0.078	1			
**V**	0.079	0.654 *	0.867 *	0.871 *	0.971 *	0.284 *	1		
**S**	0.108 *	0.804 *	0.876 *	0.796 *	0.999 *	0.075	0.975 *	1	
**A_p_**	0.149 *	0.917 *	0.910 *	0.489 *	0.913 *	−0.233 *	0.829 *	0.916 *	1

The symbol * indicates significance at *p* < 0.05; L, W, T, M, Dg, Ψ—length, width, thickness, mass, geometric mean diameter and sphericity of the seeds.

**Table 5:** Nutritional composition of rapeseeds and meal.

Parameter	Seed	Meal
Moisture, %	5.24 ± 0.13 ^a^	5.27 ± 0.12 ^a^
Ash, %	4.26 ± 0.02 ^b^	6.06 ± 0.03 ^a^
Proteins, %	24.29 ± 0.02 ^b^	35.04 ± 0.32 ^a^
Lipids, %	35.22 ± 0.40 ^a^	13.24 ± 0.03 ^b^
Total dietary fibers, %	22.95 ± 0,16 ^b^	31.80 ± 0,67 ^a^
Remaining carbohydrates, %	8.04 ± 0.43 ^b^	8.58 ± 0.78 ^a^
Energy value, kcal/100 g	492.24 ± 1.80 ^a^	342.32 ± 1.14 ^b^

Difference assessment was performed with a paired *t*-test. Values followed by ^a, b^ are statistically different at the 95% confidence level.

**Table 6:** Comparison of mineral composition for rapeseeds, meal and oil.

Mineral Composition	Seed	Meal	Oil
Lithium (Li), mg/kg	1.10 ± 0.02 ^a^	-	1.00 ± 0.01 ^b^
Beryllium (Be), mg/kg	17.90 ± 1.10 ^b^	26.30 ± 0.40 ^a^	0.40 ± 0.02 ^c^
Magnesium (Mg), mg/kg	4679.4 ± 360.5 ^a^	4194.8 ± 0.00 ^a^	8.00 ± 0.57 ^b^
Calcium (Ca), mg/kg	2139.6 ± 134.5 ^b^	3085.6 ± 69.3 ^a^	6.70 ± 0.47 ^c^
Titan (Ti), mg/kg	23.50 ± 1.90 ^a^	26.60 ± 2.50 ^a^	0.10 ± 0.00 ^b^
Chromium (Cr), mg/kg	73.00 ± 7.10 ^b^	108.5 ± 6.3 ^a^	1.00 ± 0.04 ^c^
Manganese (Mn), mg/kg	73.60 ± 6.70 ^b^	120.7 ± 9.3 ^a^	0.80 ± 0.10 ^c^
Iron (Fe-II), mg/kg	3.60 ± 0.19 ^b^	5.60 ± 0.30 ^a^	0.03 ± 0.00 ^c^
Iron (Fe-III), mg/kg	1.10 ± 0.10 ^b^	1.60 ± 0.10 ^a^	0.01 ± 0.00 ^c^
Cobalt (Co), mg/kg	1.20 ± 0.02 ^a^	1.50 ± 0.30 ^a^	0.03 ± 0.00 ^b^
Nickel (Ni), mg/kg	0.80 ± 0.02 ^b^	1.20 ± 0.00 ^a^	0.20 ± 0.00 ^c^
Copper (Cu), mg/kg	35.60 ± 2.50 ^b^	53.20 ± 0.60 ^a^	0.60 ± 0.00 ^c^
Zinc (Zn), mg/kg	47.40 ± 3.20 ^b^	80.80 ± 4.80 ^a^	0.20 ± 0.01 ^c^
Selenium (Se), mg/kg	1235.1 ± 99.7 ^a^	1396.2 ± 92.5 ^a^	2.00 ± 0.10 ^b^
Molybdenum (Mo), mg/kg	0.30 ± 0.02 ^b^	0.40 ± 0.00 ^a^	0.20 ± 0.01 ^c^
Cesium (Ce), mg/kg	356.9 ± 23.6 ^a^	501.3 ± 4.04 ^a^	63.7 ± 2.00 ^a^
Thallium (Tl), mg/kg	806.2 ± 65.9 ^a^	278.2 ± 15.0 ^a^	186.6 ± 9.75 ^a^
Total, mg/kg	9496.36	9882.71	271.28

Different superscript letters are significantly different (*p* < 0.05%) according to Tukey’s post hoc test.

**Table 7:** Fatty acid composition of rapeseeds and meal.

Parameter		Seed	Meal
Caprylic acid (C8:0)	SFA	0.41 ± 0.01 ^b^	0.65 ± 0.04 ^a^
Capric acid (C10:0)	SFA	0.36 ± 0.03 ^a^	0.23 ± 0.01 ^b^
Lauric acid (C12:0)	SFA	0.37 ± 0.02 ^a^	0.21 ± 0.00 ^b^
Myristic acid (C14:0)	SFA	0.55 ± 0.04 ^a^	0.48 ± 0.03 ^a^
Myristoleic acid (C14:1, n-5)	MUFA	0.21 ± 0.01 ^b^	1.71 ± 0.06 ^a^
Pentadecanoic acid (C15:0)	SFA	4.45 ± 0.28 ^a^	5.45 ± 0.35 ^a^
cis-10-pentadecanoic acid (C15:1, n-5)	MUFA	6.21 ± 0.03 ^a^	-
Palmitic acid (C16:0)	SFA	12.27 ± 0.87 ^a^	-
Palmitoleic acid (C16:1, n-7)	MUFA	0.52 ± 0.01 ^a^	0.65 ± 0.04 ^a^
Heptadecanoic acid (C17:0)	SFA	-	22.17 ± 0.26 ^a^
Stearic acid (C18:0)	SFA	1.08 ± 0.03 ^a^	0.47 ± 0.02 ^b^
Oleici acid + elaidic acid (C18:1, cis + trans, n-9)	MUFA	10.24 ± 0.49 ^a^	9.12 ± 0.16 ^a^
Linoleic acid + Linolelaidic acid (C18:2, cis + trans, n-6)	PUFA	44.72 ± 0.64 ^a^	39.63 ± 0.67 ^b^
γ-Linolenic acid (C18:3, n-6)	PUFA	0.64 ± 0.00 ^a^	-
α-Linolenic acid (C18:3, n-3)	PUFA	1.17 ± 0.07 ^b^	12.48 ± 0.03 ^a^
cis-11,14-eicosadienoic acid + cis-8,11,14-eicosatrienoic acid (C20:2, n-6)	PUFA	1.26 ± 0.01 ^b^	1.75 ± 0.07 ^a^
cis-11,14,17-eicosatrienoic acid (C20:3, n-3)	PUFA	0.31 ± 0.01 ^a^	0.40 ± 0.03 ^a^
Arachidonic acid (C20:4, n-6)	PUFA	0.46 ± 0.06 ^a^	-
Heneicosanoic acid (C21:0)	SFA	0.38 ± 0.01 ^a^	0.50 ± 0.04 ^a^
Eicosadienoic acid (C22:0)	SFA	0.21 ± 0.01 ^a^	0.10 ± 0.00 ^b^
Erucic acid (C22:1, n-9)	MUFA	5.57 ± 0.18 ^a^	3.75 ± 0.14 ^b^
cis-4,7,10,13,16,19-docosahexanoic acid (C22:2, n-6)	PUFA	-	0.25 ± 0.014 ^a^
cis-4,7,10,13,16,19-docosa-hexanoic + nervonic acid (C22:6, n-3 + C24:1, n-9)	PUFA	4.31 ± 0.11 ^a^	-
Tricosanoic acid (C23:0)	SFA	4.15 ± 0.03 ^a^	-
Lignoceric acid (C24:0)	SFA	0.17 ± 0.01 ^a^	-
*C18:2 w-6/C18:3 w-3*		38.22 ± 1.77	3.17 ± 0.06
*C18:1 w-9/C18:2 w-6*		0.23 ± 0.01	0.23 ± 0.00
*ΣSFAs (%)*		24.40 ± 0.56	30.25 ± 0.64
*ΣUFAs (%)*		75.62 ± 1.22	69.75 ± 0.69
*ΣMUFAs (%)*		22.75 ± 0.35	15.24 ± 0.13
*ΣPUFAs (%)*		52.87 ± 0.87	54.51 ± 0.56
*ΣSFAs/ΣUFAs*		0.32 ± 0.01	0.43 ± 0.01
*ΣPUFAs/ΣMUFAs*		2.30 ± 0.00	3.60 ± 0.01

Different superscript letters means a significant difference (*p* < 0.05%).

**Table 8:** Amino acid composition of rapeseeds and meal.

Amino Acids	Seed		Meal	
	nmol/g	%	nmol/g	%
Alanine	2081.39 ± 60.32 ^b^	5.37	2497.15 ± 58.12 ^a^	5.46
Glycine	2192.27 ± 119.30 ^b^	5.65	2886.41 ± 140.32 ^a^	6.31
α-aminobutiric acid	449.56 ± 1.69 ^a^	1.16	429.01 ± 12.17 ^a^	0.94
Valine *	1353.73 ± 33.90 ^b^	3.49	2473.47 ± 47.92 ^a^	5.41
β-aminobutiric acid	9.57 ± 0.05 ^b^	0.03	10.07 ± 0.14 ^a^	0.02
Leucine *	305.04 ± 8.16 ^a^	0.79	352.93 ± 17.06 ^a^	0.77
Isoleucine *	518.95 ± 21.26 ^a^	1.34	590.55 ± 12.22 ^a^	1.29
Threonine *	661.81 ± 0.46 ^a^	1.71	-	-
Serine	828.72 ± 5.45 ^b^	2.14	10,207.99 ± 97.43 ^a^	22.33
Proline	2761.80 ± 4.36 ^a^	7.12	1315.84 ± 29.51 ^b^	2.88
Asparagine	2931.65 ± 26.37 ^a^	7.56	2435.22 ± 51.38 ^b^	5.33
Thioproline	1991.27 ± 10.83 ^a^	5.13	1163.17 ± 21.95 ^b^	2.55
Aspartic acid	5912.94 ± 204.58 ^a^	15.24	1866.28 ± 46.35 ^b^	4.08
Methionine *	1015.11 ± 0.91 ^a^	2.62	648.43 ± 25.56 ^b^	1.42
3/4-Hidroxiproline	672.65 ± 0.26 ^a^	1.73	708.95 ± 41.18 ^a^	1.5
Phenylalanine *	444.97 ± 27.45 ^b^	1.15	735.80 ± 3.49 ^a^	1.61
Glutamic acid	7728.82 ± 116.04 ^a^	19.92	1930.72 ± 23.71 ^b^	4.22
α-aminoadipic acid	462.50 ± 1.18 ^b^	1.19	569.67 ± 2.24 ^a^	1.25
Glutamine	7728.82 ± 116.04 ^b^	3.21	8818.06 ± 4.69 ^a^	19.29
Ornithine	-	-	597.93 ± 3.11 ^a^	1.31
Glycylproline	508.17 ± 2.77 ^a^	1.31	472.08 ± 8.71 ^b^	1.03
Hidroxylysine	528.23 ± 3.18 ^a^	1.36	524.77 ± 2.24 ^a^	1.15
Proline-Hydroxyproline	508.41 ± 2.97 ^a^	1.31	446.33 ± 3.21 ^b^	0.98
Histidine *	779.33 ± 11.84 ^b^	2.01	1042.54 ± 77.08 ^a^	2.28
Lysine *	601.01 ± 2.18 ^a^	1.55	596.36 ± 68.16 ^a^	1.31
Tyrosine	458.58 ± 9.18 ^a^	1.18	462.59 ± 20.18 ^a^	1.01
Tryptophan *	532.58 ± 1.83 ^a^	1.37	534.27 ± 0.68 ^a^	1.17
Cystathionine	649.52 ± 7.04 ^a^	1.67	661.12 ± 0.81 ^a^	1.45
Cystine	662.08 ± 2.45 ^b^	1.71	732.86 ± 1.66 ^a^	1.60
Total AAs	38,795.21		45,710.55	
Essential AAs, %	16.01		15.26	
Non-essential AAs, %	83.99		84.74	

AAs: amino acids. The symbol * means essential AAs. Values are presented as mean ± standard deviation. When followed by different superscript letters (^a,b^), values are statistically different at the 95% confidence level.

**Table 9:** Functional and color properties of rapeseed meal.

Parameter	BD (g/mL)	WHC (g/g)	OHC (g/g)	EC (%)	ES (%)	FC (%)	LGC (%)	L*	a*	b*
SR	0.5942	2.92	1.29	25.65	93.33	6.47	16.00	63.29	2.14	18.29
±SD	0.004	0.18	0.05	1.19	5.77	0.56	0.00	0001	0.02	0.01

SR—rapeseed meal; SD—standard deviation; BD—bulk density; WHC—water holding capacity; OHC—oil holding capacity; EC—emulsion capacity; ES—emulsion stability; FC—foaming capacity: LGC—least gelatinization concentration L*—lightness; a*—redness; b*—yellowness.

**Table 10:** Experimental design and data values.

Run	Factors								Responses	
	Coded				Actual					
	A	B	C	D	Temperature, °C	Time, min	Amplitude, %	Solvent	TPC, mg GAE/g	DPPH, %
1	−1	−1	0	{ 1 0 }	30	10	70	water	53.41	71.50
2	1	−1	0	{ 1 0 }	50	10	70	water	111.84	77.61
3	−1	1	0	{ 1 0 }	30	20	70	water	126.88	83.00
4	1	1	0	{ 1 0 }	50	20	70	water	102.05	78.47
5	−1	0	−1	{ 1 0 }	30	15	40	water	30.00	65.55
6	1	0	−1	{ 1 0 }	50	15	40	water	46.00	80.19
7	−1	0	1	{ 1 0 }	30	15	100	water	142.32	81.70
8	1	0	1	{ 1 0 }	50	15	100	water	117.00	56.93
9	0	−1	−1	{ 1 0 }	40	10	40	water	19.00	78.47
10	0	1	−1	{ 1 0 }	40	20	40	water	104.48	75.02
11	0	−1	1	{ 1 0 }	40	10	100	water	174.00	75.00
12	0	1	1	{ 1 0 }	40	20	100	water	151.4	74.16
13	0	0	0	{ 1 0 }	40	15	70	water	47.00	83.63
14	0	0	0	{ 1 0 }	40	15	70	water	46.92	76.31
15	0	0	0	{ 1 0 }	40	15	70	water	46.92	76.14
16	−1	−1	0	{ 0 1 }	30	10	70	ethanol	47.31	44.53
17	1	−1	0	{ 0 1 }	50	10	70	ethanol	75.15	66.33
18	−1	1	0	{ 0 1 }	30	20	70	ethanol	63.35	72.22
19	1	1	0	{ 0 1 }	50	20	70	ethanol	25.00	58.00
20	−1	0	−1	{ 0 1 }	30	15	40	ethanol	7.14	40.24
21	1	0	−1	{ 0 1 }	50	15	40	ethanol	45.84	65.49
22	−1	0	1	{ 0 1 }	30	15	100	ethanol	51.54	67.00
23	1	0	1	{ 0 1 }	50	15	100	ethanol	9.41	41.08
24	0	−1	−1	{ 0 1 }	40	10	40	ethanol	35.00	50.00
25	0	1	−1	{ 0 1 }	40	20	40	ethanol	35.02	63.80
26	0	−1	1	{ 0 1 }	40	10	100	ethanol	84.29	54.00
27	0	1	1	{ 0 1 }	40	20	100	ethanol	47.77	70.00
28	0	0	0	{ 0 1 }	40	15	70	ethanol	8.00	80.00
29	0	0	0	{ 0 1 }	40	15	70	ethanol	8.00	70.00
30	0	0	0	{ 0 1 }	40	15	70	ethanol	8.00	70.00
31	−1	−1	0	{ −1 −1 }	30	10	70	methanol	112.43	82.34
32	1	−1	0	{ −1 −1 }	50	10	70	methanol	83.00	81.00
33	−1	1	0	{ −1 −1 }	30	20	70	methanol	175.72	74.00
34	1	1	0	{ −1 −1 }	50	20	70	methanol	81.06	73.09
35	−1	0	−1	{ −1 −1 }	30	15	40	methanol	83.00	70.56
36	1	0	−1	{ −1 −1 }	50	15	40	methanol	128.39	87.40
37	−1	0	1	{ −1 −1 }	30	15	100	methanol	141.21	78.55
38	1	0	1	{ −1 −1 }	50	15	100	methanol	28.14	49.54
39	0	−1	−1	{ −1 −1 }	40	10	40	methanol	118.3	89.07
40	0	1	−1	{ −1 −1 }	40	20	40	methanol	193.63	82.34
41	0	−1	1	{ −1 −1 }	40	10	100	methanol	135.89	81.50
42	0	1	1	{ −1 −1 }	40	20	100	methanol	114.21	70.00
43	0	0	0	{ −1 −1 }	40	15	70	methanol	67.00	86.00
44	0	0	0	{ −1 −1 }	40	15	70	methanol	68.00	85.70
45	0	0	0	{ −1 −1 }	40	15	70	methanol	66.79	86.00

**Table 11:** ANOVA results for model fitting (*** *p* < 0.001; ** *p* < 0.01; * *p* < 0.05). Coefficients of regression that explain the effect of the variables and their interactions.

Factors	TPC	DPPH
R^2^	0.9469	0.9111
Adjusted R^2^	0.9134	0.8551
F value	28.30	16.27
*p* value	<0.0001	<0.0001
Lack of Fit	0.2857	0.3560
b_0_	+44.74	+79.31
b_1_	−7.56 *	−0.67
b_2_	+7.12 *	+0.95
b_3_	+14.64 ***	−2.03 *
b_4_	+12.51 ***	+3.95 ***
b_5_	−41.12 ***	−10.79 ***
b_6_	−17.89 ***	−3.85 **
b_7_	−23.38 ***	−11.37 ***
b_8_	+10.59 **	−0.40
b_9_	+5.82 **	+1.53
b_10_	−20.14 ***	+0.003
b_11_	+8.70 **	+0.061 ***
b_12_	−15.95 **	+5.20 ***
b_13_	+33.51 ***	+0.60 **
b_14_	−5.89 ***	+3.60 **
b_15_	+5.72	−7.03 ***
b_16_	+37.64 ***	−0.44
b_17_	+18.71 **	−6.93 ***

**Table 12:** Phenolic compounds in rapeseed meal.

Phenolic Acids	Results, mg/kg
4-Hydroxybenzoic acid	155.49 ± 5.80
Vanillic acid	2496.71 ± 46.88
Caffeic acid	1885.43 ± 54.27
Chlorogenic acid	1891.18 ± 19.53
p-cumaric acid	30.53 ± 0.92
Rosmarinic acid	354.09 ± 11.29
Myricetin	1966.51 ± 179.52
Luteolin	17.89 ± 0.64
Quercetin	460.45 ± 30.72
Kaempferol	14.50 ± 0.92
Total	9272.28

## Data Availability

The original contributions presented in the study are included in the article, further inquiries can be directed to the corresponding authors.
